# Development of Coarse-Grained Liquid-Crystal Polymer Model with Efficient Electrostatic Interaction: Toward Molecular Dynamics Simulations of Electroactive Materials

**DOI:** 10.3390/ma11010083

**Published:** 2018-01-06

**Authors:** Kenji Tagashira, Kazuaki Z. Takahashi, Jun-ichi Fukuda, Takeshi Aoyagi

**Affiliations:** 1 Research Association of High-Throughput Design and Development for Advanced Functional Materials, Central 2, 1-1-1 Umezono, Tsukuba-shi, Ibaraki 305-8568, Japan; k-tagashira@admat.or.jp; 2 Panasonic Corporation, 3-4 Hikaridai, Seika-cho, Soraku-gun, Kyoto 619-0237, Japan; 3 Research Center for Computational Design of Advanced Functional Materials, National Institute of Advanced Industrial Science and Technology (AIST), Central 2, 1-1-1 Umezono, Tsukuba-shi, Ibaraki 305-8568, Japan; aoyagi.t@aist.go.jp; 4 Department of Physics, Faculty of Science, Kyushu University, 744 Motooka, Nishi-ku, Fukuoka-shi, Fukuoka 819-0395, Japan; fukuda.jun-ichi@phys.kyushu-u.ac.jp

**Keywords:** liquid-crystal polymer, coarse-grained molecular dynamics, soft-core Gay-Berne model, smeared charge

## Abstract

Liquid-crystal polymers (LCPs) are well known materials for functional sensor and actuators, because of their high-responsiveness to an electric field. Owing to their complex physical nature, however, the prediction of the functions of LCPs is a challenge. To attack this problem from a molecular point of view, a simulation study is a promising approach. In this work, for future applications of molecular dynamics simulations to problems involving an electric field, we develop an LCP model which consists of coarse-grained mesogenic molecules and smeared charges. For the smearing function of the electrostatic force, the Gauss error function is introduced. This smearing is optimized to attain a reasonable accuracy for phase transition phenomena of liquid crystal while numerical instabilities arising from the singularity of the Coulomb potential are circumvented. For swelling systems, our LCP model exhibits the characteristics of both liquid crystals and unentangled polymer chains; orientational order of the mesogenic units and Rouse-like relaxation dynamics. Our coarse-grained LCP model successfully incorporates electric charges and dipoles and is therefore applicable to problems concerning an electric field.

## 1. Introduction

Liquid-crystal polymers (LCPs) consist of open chain compounds containing mesogenic units. It is well known that LCPs exhibiting liquid crystal phases show high-responsiveness to external stimuli such as an electric and magnetic fields, and irradiated light [1,2,3,4]. Cross-linked LCPs, referred to as liquid crystal elastomers (LCEs), liquid crystal networks (LCNs) and so on, have elastic characteristics, while maintaining the above-mentioned responsiveness that, in combination with cross-linked network, gives rise to macroscopic deformations [5,6,7,8,9,10]. This unique property of cross-linked LCPs indicates their applicability to sensing devices as well as actuator devices [11,12,13]. The development of these devices, however, is a challenge because the molecular-level mechanics of the deformation is not understood enough. The complex structures, dynamics, and physical properties of LCPs at different time and length scales over a wide range are closely related. In other words, a slight modification of atomistic-scale properties such as molecular architectures can drastically change meso- and macro-scale characteristics of LCPs; the understanding of the behavior of LCPs is a typical multiscale problem. Therefore, to attack this critical problem and predict physical properties of LCPs, simulational studies from molecular point of view should be introduced. Molecular models and simulations for LCPs have been developed and performed over ten years [14,15,16,17].

Skačej et al., developed an off-lattice coarse-grained LCP model using the soft-core Gay-Berne (SCGB) ellipsoidal potential, and carried out Monte-Carlo simulations under simplified body-force-like electric field conditions [16]. They observed the reorientation of ellipsoids induced by the external field, and the resulting deformation of LCPs. Whitmer et al., developed a flexibly bonded Gay-Berne (GB) model, and carried out molecular dynamics (MD) simulations [17]. A polydomain-monodomain transition of LCPs was observed under uniaxial strain. These previous studies indicate that coarse-grained simulations can provide a promising tool for the investigation of mesoscopic deformations of LCPs induced by external stimuli. It is important to note, however, that molecular simulations for macroscopic deformation of LCPs are still beyond the capability of current computational resources. Thus, hierarchical linkage between molecular simulations and macroscopic methods, such as the finite element method (FEM), is required. In FEM, electric-field-induced deformations can be computed using the piezoelectric tensor analysis [18]. For the linkage between two simulations with different time and length scales, therefore, the piezoelectric tensor should be directly calculated from molecular simulations. To enable the direct calculation, the exact expression of the dielectric character of molecular systems is necessary. Placing point charges on the coordinate of atoms is one simple and efficient way to express the dielectric character of molecular systems, and is a normal way for all-atomistic molecular simulations. For coarse-grained models, however, placing point charges has difficulty because of the bad combination between softened interaction for coarse-grained models and Coulomb interaction for point charges. Namely, the Coulomb potential has a singularity at zero distance of interaction while the softened potential does not. To circumvent the problem, a smearing function which removes the singularity can be introduced for the Coulomb potential. This “smeared charge” has been used for several simple coarse-grained models [19,20], but has not been developed for complex coarse-grained LCP models.

In this work, toward future molecular simulations of LCPs, we develop a coarse-grained LCP model. To enable the direct calculation of piezoelectric tensor, the dipole moment on a mesogenic unit is expressed using a pair of point charges. The Coulomb force for point charges is smeared using the Gauss error function. With using this smeared charge, the coarse-grained molecular simulations are stabilized while maintaining reasonable accuracy to investigate the phase transition phenomena of LCPs qualitatively. We perform MD simulations using this model for LCP swelling systems without the effect of an external field. The results show that our model exhibits the characteristics of liquid-crystalline orientational order and Rouse-like relaxation dynamics of unentangled polymer chains. We conclude that our coarse-grained LCP model has the possibility of hierarchically linking molecular and macroscale simulations.

## 2. Methodology

### 2.1. Coarse-Grained LCP Model

Our coarse-grained LCP model can be thought of as a modified version of the model of Skačej et al. [16,21]. The main difference from their original model is the introduction of point charges for the direct calculation of the piezoelectric tensor. A mesogenic dipole moment is represented by a pair of charges ±q placed on the SCGB ellipsoid. For stabilizing coarse-grained molecular simulations, the electrostatic interaction between point charges should be carefully treated. The Coulomb potential cannot be used as is together with soft-core potentials, because the former has a singularity at zero distance of interaction while the latter does not. Without a proper treatment for point charges, simulations become unstable. The introduction of a smeared charge is one of the possible ways to handle this problem. Several types of smeared charges have been introduced for dissipative particle dynamics simulations [19,20]. In this work, the Coulomb force between point charges *Q* and *q* is modified by introducing the Gauss error function in the following manner:

(1) $$F_{\mathrm{ele}}=-\nabla U_{\mathrm{ele}}=k\frac{qQ}{r^{2}}\frac{r}{r}\mathrm{erf}\left(\alpha r\right),$$

where $U_{\mathrm{ele}}$ is the electrostatic potential, *k* is Coulomb constant that will be set to unity in our simulations, r is the vector representing the relative distance between two charges, r=|r|, and α in the error function concerns the strength of smearing. The following four conditions are desirable for the smearing function. (i) The function is continuous at any *r*. (ii) The singularity of Coulomb force at r=0 can be removed. (iii) The shape of the original Coulomb force is kept, except near r=0. (iv) The implementation and computation are easy. Since the Gauss error function erf(αr) with large α satisfies the above four conditions, it is ideal for a modified Coulomb force and useful for a wide variety of coarse-grained models.

**Figure 1:**
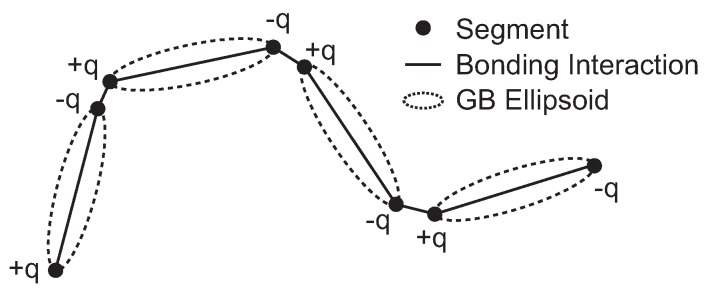
Schematic illustration of our coarse-grained liquid-crystal polymer (LCP) model.

The implementation for mesogenic ellipsoid follows that of the course-grained MD program COGNAC [22,23]. As shown in Figure 1, a GB ellipsoid is represented by two segments that are placed at the ends of its principal axis. For simplifying the computation, a pair of point charges is placed on the two segments belonging to the same ellipsoidal unit. The segments are bonded so that they constitute straight chain polymers. COGNAC is developed as a general purpose coarse-grained molecular dynamics package to adopt various kinds of coarse-grained model and to be able to introduce the user-defined potential function. Thus, the coarse-grained model used in this study can be easily implemented.

For the intermolecular interaction between ellipsoids, SCGB potential [16] is used. The original GB potential is a well-known anisotropic interaction potential which has been widely used to study various kinds of liquid crystal phases and systems [24]. The GB potential energy $U_{ij}^{\mathrm{GB}}$ is expressed as follows:

(2) $$U_{ij}^{\mathrm{GB}}=4\epsilon_{ij}\left\{{\left[\frac{\sigma_{s}}{r_{ij}-\sigma_{ij}+\sigma_{s}}\right]}^{12}-{\left[\frac{\sigma_{s}}{r_{ij}-\sigma_{ij}+\sigma_{s}}\right]}^{6}\right\},$$

where $r_{ij}$ represents the vector between the centers of the mass for a pair of ellipsoids, *i* and *j*, $r_{ij}=\left|r_{ij}\right|$ and ${\hat{r}}_{ij}=r_{ij}/r_{ij}$. The unit vector $u_{i}$ is along the long axis of the ellipsoid *i*. The length parameter for the ellipsoid pair $\sigma_{ij}$ is given by

(3) $$\sigma_{ij}=\sigma_{0}\left\{1-\frac{\chi }{2}\left[\frac{{\left(u_{i}\cdot {\hat{r}}_{ij}+u_{j}\cdot {\hat{r}}_{ij}\right)}^{2}}{1+\chi (u_{i}\cdot u_{j})}+\frac{{\left(u_{i}\cdot {\hat{r}}_{ij}-u_{j}\cdot {\hat{r}}_{ij}\right)}^{2}}{1-\chi (u_{i}\cdot u_{j})}\right]\right\},$$

where

(4) $$\chi =\frac{\kappa^{2}-1}{\kappa^{2}+1}.$$

Here, $\kappa =\sigma_{e}/\sigma_{s}$, with $\sigma_{e}$ and $\sigma_{s}$ denoting the range parameter for the end-to-end and side-by-side configurations, respectively. The anisotropic energy parameter $\epsilon_{ij}$ is written as

(5) $$\epsilon_{ij}=\epsilon_{0}{\left(\epsilon_{ij}^{\prime }\right)}^{\mu }{\left(\epsilon_{ij}^{\prime \prime }\right)}^{\nu }.$$

Here, $\epsilon_{0}$ is a characteristic GB interaction strength, μ and ν are dimensionless parameters, and

(6) $$\begin{array}{c} \epsilon_{ij}^{\prime }=1-\frac{\chi^{\prime }}{2}\left[\frac{{\left(u_{i}\cdot {\hat{r}}_{ij}+u_{j}\cdot {\hat{r}}_{ij}\right)}^{2}}{1+\chi^{\prime }\left(u_{i}\cdot u_{j}\right)}+\frac{{\left(u_{i}\cdot {\hat{r}}_{ij}-u_{j}\cdot {\hat{r}}_{ij}\right)}^{2}}{1-\chi^{\prime }\left(u_{i}\cdot u_{j}\right)}\right], \end{array}$$

(7) $$\begin{array}{c} \epsilon_{ij}^{\prime \prime }={\left[1-\chi^{2}{\left(u_{i}\cdot u_{j}\right)}^{2}\right]}^{-1/2}, \end{array}$$

(8) $$\begin{array}{c} \chi^{\prime }=\frac{\kappa^{\prime \;1/\mu }-1}{\kappa^{\prime \;1/\mu }+1}, \end{array}$$

where $\kappa^{\prime }=\epsilon_{s}/\epsilon_{e}$, with $\epsilon_{s}$ and $\epsilon_{e}$ denoting the potential well depth in the side-by-side and end-to-end configurations, respectively, and $\sigma_{0}$ is set to $\sigma_{s}$ as a characteristic van der Waals diameter.

In the SCGB potential, the strong core repulsion in the original GB potential is replaced by a weaker linear repulsion. The SC potential energy $U_{ij}^{\mathrm{SC}}$ is written as

(9) $$U_{ij}^{\mathrm{SC}}=m\left(r_{ij}-\sigma_{ij}\right).$$

Here, *m* stands for the potential slope and $r_{ij}$ and $\sigma_{ij}$ are already defined above. The GB and SC potentials are merged using a sigmoidal logistic function $f_{ij}=1/\left\{1+\exp\left[n\left(\sigma_{ij}-r_{ij}\right)\right]\right\}$ so that the resulting potential reads

(10) $$U_{ij}^{\mathrm{GBSC}}=\left(1-f_{ij}\right)U_{ij}^{\mathrm{GB}}+f_{ij}U_{ij}^{\mathrm{SC}}.$$

In this study, the van der Waals diameter $\sigma_{0}$, GB interaction strength $\epsilon_{0}$ and mass of ellipsoids $m_{0}$ are set to unity, and $\sigma_{s}$ is set equal to $\sigma_{0}$. The potential parameters used are μ=1, ν=3, $\sigma_{e}/\sigma_{s}=3$, $\epsilon_{s}/\epsilon_{e}=5$, $m=-70\epsilon /\sigma_{0}$, $n=-100/\sigma_{0}$. The cutoff distance is $r_{c}=4.2\sigma_{0}$.

For the bonding potential between adjacent segments *k* and k+1, a harmonic potential is introduced as below:

(11) $$U_{k}^{\mathrm{bond}}=\frac{1}{2}k{\left(r-r_{0}\right)}^{2}.$$

Here, *k* is the spring constant, *r* is the bond length and $r_{0}$ is the equilibrium bond length. This simulation system possesses two kinds of bonding, inter- and intra-ellipsoid bonding, since the GB ellipsoid is defined by two segments. For inter-ellipsoid bonding, $r_{0}=0.15\sigma_{0}$ and $k=100\epsilon_{0}/\sigma_{0}^{2}$ are adopted. On the other hand, for intra-ellipsoid bonding, $r_{0}=\kappa \sigma_{0}=3.0\sigma_{0}$ and $k=10,000\epsilon_{0}/\sigma_{0}^{2}$. We choose the spring constant for the intra-ellipsoid bonding much larger than that for the inter-ellipsoid bonding so as to prevent the deviation of the segment location from the end of the ellipsoids.

### 2.2. Simulation Detail

Our MD simulation system is a cubic cell of volume *V* including 121 LCP molecules each made up of 30 GB particles, and 3630 GB monomer particles. Therefore N=7260 GB particle are in our system in total, and the number of GB particles constituting LCP molecules is equal to that of GB monomer particles. The dimensionless density is $\rho^{*}\equiv N\sigma_{0}^{3}/V=0.3$. The equations of motion are solved by the velocity Verlet algorithm [25] with a time step of $\Delta t^{*}={\left(\epsilon_{0}/\sigma_{0}^{2}m_{0}\right)}^{1/2}\Delta t=0.005$. In the simulations, the NVE ensemble is employed and velocities of mass points are scaled every 100 time steps to adjust temperature. The simulation is started with randomly oriented configuration at high temperature $T^{*}\equiv k_{B}T/\epsilon_{0}=10.0$, and the temperature is decreased stepwise with the interval $\Delta T^{*}=0.5$. At each temperature step, at least $10^{6}$ time steps of the calculation are carried out so as to obtain relaxed states. The relaxed configuration is utilized as the initial structure of the next temperature step. For the evaluation of the relaxation time, at least $10^{7}$ time steps of calculations are carried out, and in addition, 5 samples with different initial configurations are averaged. All of the simulations are done by COGNAC [22].

## 3. Results and Discussions

**Figure 2:**
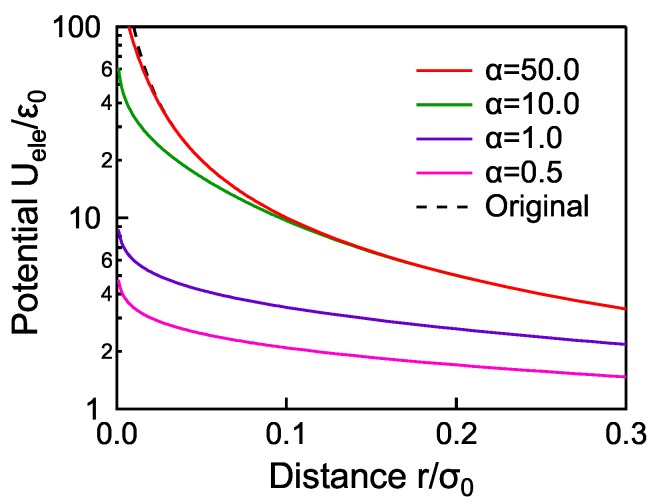
The potential profiles for the smeared electrostatic potentials with the strengths α=0.5,1.0,10.0,50.0. The original 1/r potential is given by a dashed line.

The smearing strength of the electrostatic potential is adjustable by the parameter α in Equation (1). Figure 2 shows the potential profiles $U_{\mathrm{ele}}\left(r\right)$ for various values of α, where qQ=−1. The original unsmeared potential 1/r is denoted by a dashed line. The potential is smeared more strongly with decreasing α, but the effect of the smearing should be as small as possible due to its artificiality. On the other hand, a certain level of smearing is essential for the stability of the simulations. In order to find an appropriate value of α, we consider the influence of α on the phase transition behaviors. The phase transition behavior is evaluated by the temperature dependence of the order parameter *S* defined as

(12) $$S=\frac{1}{2N_{\mathrm{LCP}}}\sum_{i}^{N_{\mathrm{LCP}}}\langle 3{\left(n\cdot u_{i}\right)}^{2}-1\rangle ,$$

where $N_{\mathrm{LCP}}$ represents the number of GB particles composing the LCPs (not including the solvent GB particles), and the director n denotes the average orientation direction of GB particles composing the LCPs.

**Figure 3:**
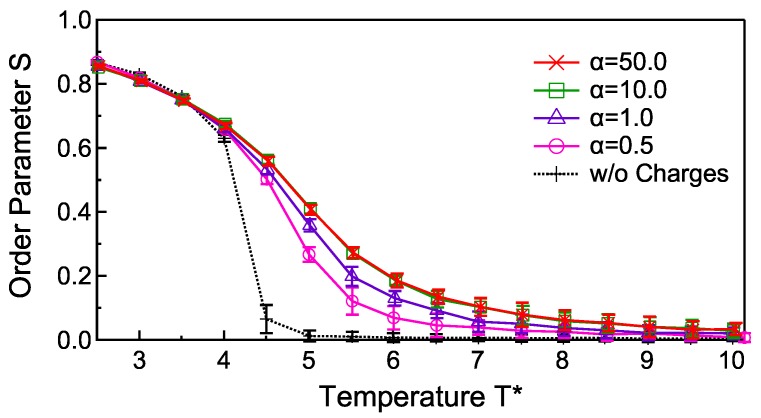
Temperature dependences of the order parameter for the smeared electrostatic potentials with the strengths α=0.5,1.0,10.0,50.0. The order parameter without the electrostatic potential is given by a dashed line.

Figure 3 shows the temperature dependence of the order parameter at various smearing strengths, α=0.5,1.0,10.0,50.0, for q=0.3. For α>50.0, the calculation collapses. For the system without electric charges (dashed line), the drastic temperature variation of the order parameter (nematic-isotropic phase transition) is observed around $T^{*}=4.5$. With increasing α, the phase transition behaviors are smoothed and the transition temperatures are shifted higher. The two plots for α=10.0 and 50.0 are, however, completely overlapped, which means that the introduced error function does not affect the phase transition behavior at α>10.0. In this region of α, the potential profiles are indistinguishable from the original 1/r potential at $r/\sigma_{0}>0.1$ as shown in Figure 2. From these facts, we conclude that the smearing of the electrostatic potential at $r/\sigma_{0}<0.1$ does not affect the phase transition behavior, whereas it stabilizes the calculation. In other words, the potential profile should be set to 1/r at $r/\sigma_{0}>0.1$ for preventing artificial effects on the relaxation state, and smeared enough at $r/\sigma_{0}<0.1$ for stable calculations. Therefore, we adopt α=10.0 in the simulations below.

There are two possible reasons for the smoothed phase transition behavior by introducing the electrostatic potentials. One is the influence of finite system size. The phase transition behaviors are confirmed to be little affected when the linear dimension of the system is doubled, and therefore the finite size effect is not likely to be the major cause of the smoothed phase transition behavior in the presence of electrostatic interactions. The other is the alignment of ellipsoids induced by the electrostatic potential. The ellipsoids with large spontaneous polarizations are stabilized strongly due to the head-to-tail dipolar interactions that affect the phase transition temperature [26,27]. Moreover, when an electric field is applied throughout the nematic-isotropic phase transition, the temperature dependence of the order parameter is smoothed and the (smoothed) transition is shifted to higher temperature due to the electric-field-induced stabilization of the liquid crystal alignment [28]. In our model, the alignment of ellipsoids with strong polarization also generates global polarization, which is a situation similar to that of a system polarized by external electric fields. By introducing the electrostatic interaction, the nematic phase is stabilized especially at $T^{*}>4.5$, where the isotropic phase appears in the absence of electrostatic interactions. As a result, the phase transition is shifted to higher temperature and smoothed by introducing the electrostatic charges.

**Figure 4:**
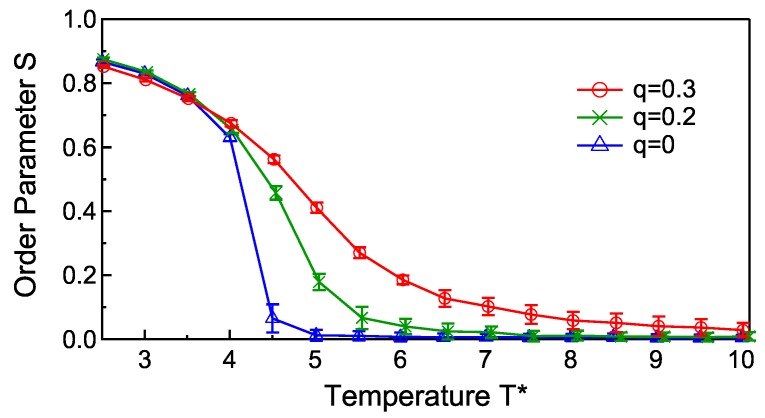
Temperature dependence of the order parameter for q=0,0.2,0.3, where α=10.0.

Figure 4 shows the phase transition behaviors for several values of the electric charges, q=0,0.2,0.3, where α=10.0. For q>0.3, the calculation is extremely unstable. With increasing the charge, the phase transition is smoothed and shifted to a higher temperature due to the electrostatic stabilization of the ellipsoid alignment stated above.

**Figure 5:**
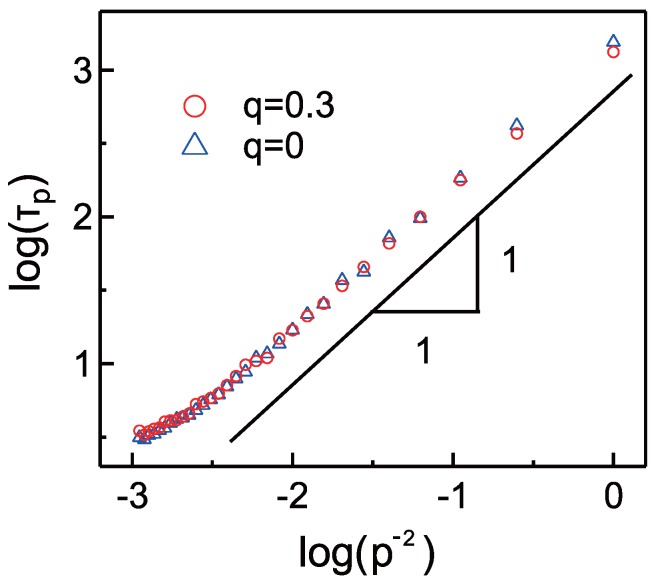
The mode index *p* dependence of the relaxation time for the autocorrelation function of the normal mode $C_{p}$ upon the isotropic state, $T^{*}=6.0$.

The understanding of the relaxation dynamics is crucial for the elucidation of the mechanical characteristics of LCPs. For this purpose, we analyze the autocorrelation of normal modes $C_{p}$ at the isotropic phase ($T^{*}=6.0$), which is written as [29],

(13) $$C_{p}\left(t\right)=\left|\frac{\langle X_{p}\left(t\right)X_{p}\left(0\right)\rangle }{\langle X_{p}\left(0\right)X_{p}\left(0\right)\rangle }\right|=\exp\left(-t/\tau_{p}\right),$$

with

(14) $$X_{p}\left(t\right)=\frac{1}{N_{\mathrm{LCP}}}\sum_{i}^{N_{\mathrm{LCP}}}r_{i}\left(t\right)\cos\frac{p\pi (i-1)}{N_{\mathrm{LCP}}-1}-\frac{1}{2N}\left\{r_{1}\left(t\right)+{\left(-1\right)}^{p}r_{{}_{N_{\mathrm{LCP}}}}\left(t\right)\right\},$$

where *p* is the index of the normal mode, $\tau_{p}$ is the relaxation time of $C_{p}$ and $r_{i}$ represents the position of the center of the mass in the *i*-th ellipsoid. Figure 5 shows the mode index *p* dependence of the relaxation time $\tau_{p}$ for q=0 and 0.3. We find that the relaxation time $\tau_{p}$ do not depend on the strength of the electrostatic interaction in the isotropic phase. Interestingly, the relaxation of LCPs exhibits the Rouse behavior, where $\tau_{p}$ is proportional to $p^{-2}$ [30]. This result suggests that the LCPs in our coarse-grained model are unentangled and flexible enough in spite of the rigid nature of the GB ellipsoids. This is partly because the swelling monomers improve the mobility of LCP chains and disturb the inter-molecular interaction. Furthermore, our LCP chains are likely to be too short to induce entanglement; more than around 100 monomers per chain are needed for the entanglement in the case of the typical bead-spring model [29]. Therefore, we speculate that the entanglement is observable in our LCP model by decreasing the solvent concentration and preparing longer chains.

## 4. Conclusions

We developed an LCP model which uses coarse-grained molecules and smeared electrostatic charge toward the prediction of electrostrictive dynamics. For the smearing function of the electrostatic force, we introduced the Gauss error function. The smearing strength was optimized so as to attain a reasonable accuracy for phase transition phenomena of liquid crystals. The nematic-isotropic phase transition was smoothed and shifted to higher temperature due to the electrostatic interaction. This is because the strong polarizations on the aligned ellipsoids generate a global polarization, which stabilize the nematic phase. In addition, the relaxation analysis for the autocorrelation of normal modes revealed that our model exhibited unentangled polymer characteristics. In other words, we confirmed that our LCP model with smeared charges exhibited both characteristics of liquid crystals and polymers. Thus, we succeeded in the development of an LCP model that can react to the electric field, and our model will be useful for predicting the mechanical properties of LCPs induced by the alignment of the mesogenic units. In further studies, the piezoelectric tensor will be calculated by this model, which will enable the hierarchical linkage between microscopic modeling and macroscopic electrostrictive simulations. We believe that this linkage for the prediction of the macroscopic functions of LCPs leads to successful molecular designing for the development of LCP sensors and actuators.
